# Antiviral Peptides Delivered by Chitosan-Based Nanoparticles to Neutralize SARS-CoV-2 and HCoV-OC43

**DOI:** 10.3390/pharmaceutics15061621

**Published:** 2023-05-30

**Authors:** Avinash Mali, Gianluigi Franci, Carla Zannella, Annalisa Chianese, Shubaash Anthiya, Ana M. López-Estévez, Alessandra Monti, Anna De Filippis, Nunzianna Doti, María José Alonso, Massimiliano Galdiero

**Affiliations:** 1 Department of Experimental Medicine, University of Campania “Luigi Vanvitelli”, 80138 Naples, Italy; sksonavinash@gmail.com (A.M.); carla.zannella@unicampania.it (C.Z.); annalisa.chianese@unicampania.it (A.C.); anna.defilippis@unicampania.it (A.D.F.); 2 Department of Medicine, Surgery and Dentistry, “Scuola Medica Salernitana”, University of Salerno, 84081 Baronissi, Italy; gfranci@unisa.it; 3 Center for Research in Molecular Medicine and Chronic Diseases (CIMUS), Health Research Institute of Santiago de Compostela (IDIS), Department of Pharmacy and Pharmaceutical Technology, School of Pharmacy, University of Santiago de Compostela, 15782 Santiago de Compostela, Spain; shubaash.anthiyaramamoorthi@usc.es (S.A.); anamarialopez.estevez@usc.es (A.M.L.-E.); 4 Institute of Biostructures and Bioimaging (IBB), National Research Council (CNR), 80131 Naples, Italy; alessandra.monti@ibb.cnr (A.M.); nunzianna.doti@cnr.it (N.D.); 5 CIRPEB, Centro Interuniversitario di Ricerca sui Peptidi Bioattivi, 80134 Naples, Italy

**Keywords:** nanoparticles, peptides, SARS-CoV-2, chitosan, coronavirus

## Abstract

The COVID-19 pandemic has made it clear that there is a crucial need for the design and development of antiviral agents that can efficiently reduce the fatality rate caused by infectious diseases. The fact that coronavirus mainly enters through the nasal epithelial cells and spreads through the nasal passage makes the nasal delivery of antiviral agents a promising strategy not only to reduce viral infection but also its transmission. Peptides are emerging as powerful candidates for antiviral treatments, showing not only a strong antiviral activity, but also improved safety, efficacy, and higher specificity against viral pathogens. Based on our previous experience on the use of chitosan-based nanoparticles to deliver peptides intra-nasally the current study aimed to explore the delivery of two-novel antiviral peptides making use of nanoparticles consisting of HA/CS and DS/CS. The antiviral peptides were chemically synthesized, and the optimal conditions for encapsulating them were selected through a combination of physical entrapment and chemical conjugation using HA/CS and DS/CS nanocomplexes. Finally, we evaluated the in vitro neutralization capacity against SARS-CoV-2 and HCoV-OC43 for potential use as prophylaxis or therapy.

## 1. Introduction

The COVID-19 pandemic is an ongoing pandemic caused by the severe acute respiratory syndrome coronavirus 2 (SARS-CoV-2) [1]. It has affected over 700 million individuals in more than 235 countries and has been responsible for the deaths of over six million people worldwide [2,3]. The world has been affected by the COVID-19 pandemic since the beginning of 2020, impacting the pace, form, and essence of daily life [4]. The pandemic was exacerbated by the scarcity of effective treatments and the absence of vaccines [5]. Coronaviruses (CoVs) have a single-stranded RNA genome with a spike glycoprotein on the surface and infect both animals and humans [6]. While vaccination is a useful method to prevent viral infections, the availability, widespread use, and efficacy of vaccines in eradicating the disease remain uncertain [7,8].

The increasing prevalence of viral resistance and the frequent emergence of viral infections pose significant challenges for the use of antiviral agents. In response to the COVID-19 pandemic, the scientific community is working to develop new antiviral treatments and effective vaccines. Nanoparticle-enabled delivery systems with antiviral agents may offer a valuable solution, as their structure, composition, and functional properties can be easily controlled [9,10,11,12,13].

Antiviral peptides (AVPs) have short and simple amino acid sequences and offer potential as broad-spectrum antivirals [9,14]. They work by blocking viral infections through binding and preventing the virus from accessing its host cell receptors or by attaching to the virus itself and blocking the ability to bind to receptors [15,16]. They can also act as inhibitors of viral proteases, preventing the formation of active viral proteins [17,18,19,20], or as inhibitors of viral polymerases, disrupting the viral replication process [21]. The antiviral bioactive peptides need to be properly formulated depending on the route of administration and their target site [22,23]. In particular, the intranasal route enables the direct targeting of the respiratory tract, which is the primary site of viral entry for respiratory viruses such as SARS-CoV-2 and HCoV-OC43. This makes it an attractive alternative for the treatment and prevention of respiratory viral infections. Our research team pioneered the development of nanoparticles made of chitosan alone or in combination with other polymers and explored their potential for the nasal administration of peptides and proteins [24,25,26,27,28]. This potential was attributed to the capacity of these nanoparticles to load large amounts of peptide molecules and also to their favorable interaction with the nasal mucosa.

Based on this background experience, the present study was aimed to evaluate the effectiveness of intranasal delivery of two novel AVPs using biocompatible biopolymers such as HA and CS. Recently, our group demonstrated that two small peptides, each consisting of only three amino acid residues (TLH and VFI), exhibited an antiviral activity against the two beta-coronaviruses used in the study, i.e., SARS-CoV-2 and HCoV-OC43; meanwhile they reported no effect against alpha-coronavirus [29]. The two peptides were derived from two recurring nucleotide strings present in different human pathogens, including SARS-CoV-2. Then we synthesized the two derived peptides (hereafter indicated as pep 1 and pep 2) and demonstrated that they were able to bind to the receptor binding domain (RBD) of the spike protein, as indicated by molecular docking and biochemical studies. The peptides had no cytotoxicity nor hemolysis activity, and a moderate stability in human serum. We interestingly observed that both the peptides interfered with SARS-CoV-2 infection: pep 1 (amino acids sequence: VFI) bound to the subunit 2 (S2) of the spike (S) protein, while pep 2 (amino acids sequence: TLH) bound to the subunit 1 (S1). On the other side, only pep 1 exhibited an antiviral effect against HCoV-OC43 infection, probably since it bound to an external site in the S protein, differently from pep 2, recognizing an internal pocket. 

The association of AVPs to the nanoparticles was performed through physical entrapment or chemical conjugation [30,31]. The most promising formulations were evaluated for in vitro neutralization of SARS-CoV-2 and HCoV-OC43 infections. 

## 2. Materials and Methods

### 2.1. Materials

Protected amino acids, coupling agents (1-[Bis(dimethylamino)methylene]-^1^H-1,2,3-triazolo[4,5-b]pyridinium 3-oxide hexafluorophosphate, Hexafluorophosphate azabenzotriazole tetramethyl uronium, HATU, (Oxyma), and N-9-Fluorenylmethyloxycarbonyl (Fmoc)-Rink amide amide aminomethyl-polystyrene (AM) resin used for peptide synthesis were purchased from IRIS Biotech GmbH (Marktrewitz, Germany). Solvents, including acetonitrile (CH_3_CN), dimethylformamide (DMF), trifluoroacetic acid (TFA), sym-collidine, diisopropylethylamine (DIPEA), piperidine, tri-isopropyl silane (TIS), were purchased from Merck (Milan, Italy). Chitosan (CS, hydrochloride salt, molecular weight (MW) 42.7 KDa and 88% deacetylation degree) was obtained from HMC+ (Halle, Germany). Dextran sulfate (DS), (sodium salt, MW 8 KDa) was purchased from. (Toronto, ON, Canada), 4-maleimidobutyric acid, N-hydroxysuccinimide (NHS), diisopropyl carbodiimide (DIC), and 1-[3-dimethylaminopropyl]-3-ethyl carbodiimide (EDC) from Sigma Aldrich (St. Louis, MI, USA) and Spectra/Por[R] dialysis tubing membranes from VWR (Radnor, Pennsylvania, United States). Tris(2-carboxyethyl) phosphine hydrochloride (TCEP) was acquired from Alfa Aesar (Kandel, Germany). Sodium hyaluronate (HA) 40 kDa (HA40) and N-(2-aminoethyl) maleimide trifluoroacetate salt were obtained from Lehvoss (Hamburg, Germany) and Phylcare (Makati, Manila, Philippines), respectively.

### 2.2. Chemical Synthesis and Purification of Peptides

Following the Fmoc method and employing HATU-collidine as coupling reagents, peptides were synthesized on solid-phase on Rink-4-methylbenzhydrylamine (MBHA) resin (loading 0.4–0.8 mmol/g) [32]. For peptide cleavage, a solution of TFA, TIS, and water (95:2.5:2.5, *v*/*v*/*v*) were stirred for three hours at room temperature. The peptides were then precipitated using cold diethyl ether, the pellets were reconstituted in a 75:25 *v*/*v* solution of H_2_O and CH_3_CN and lyophilized. Reverse-phase HPLC (RP-HPLC) was used to purify the crude peptides using a WATERS 2545 preparative system (Waters, Milan, Italy) outfitted with a WATERS 2489 UV/Vis detector. Using a Jupiter C18 (5 μm, 150 × 21.2 mm ID) column, the purification step was carried out at 15 mL/min while monitoring the absorbance at 214 nm. A linear gradient of 0.1% TFA in CH_3_CN from 5% to 70% was applied for 15 min.

### 2.3. Peptide Characterization

The identity of peptides was assessed by liquid chromatography-mass spectrometry analysis (LC-MS) using an electrospray ionization time-of-flight mass spectrometry (ESI-TOF-MS) Agilent 1290 Infinity LC System coupled to an Agilent 6230 TOF-LC/MS System (Agilent Technologies, Cernusco Sul Naviglio, Italy). The LC Agilent 1290 LC module was coupled with a photodiode array (PDA) detector and a 6230 TOF-MS detector, along with a binary solvent pump degasser, a column heater, and an autosampler. LC-MS characterization of peptides was performed using a C18 Waters XBridge column (3 μm, 4.6 × 5.0 mm), applying a linear gradient of CH_3_CN/0.05% TFA in water/0.05% TFA from 5 to 70% in 15 min, at a flow rate of 0.2 mL/min. The relative purity of peptides was calculated as the ratio of the peak area of the target peptide and the sum of areas of all detected peaks from the UV chromatograms at 210 nm. The purity of all tested peptides was over 95%.

### 2.4. Screening of Blank Nanoparticles (NPs)

For CS/DS NPs and CS/HA NPs, mass ratios of 1:0.5 to 1:3 and 1:0.5 to 1:4 were screened respectively and in CS/DS NPs; an equal volume (0.5 mL) of aqueous solution of CS (1 mg/mL) was added to an aqueous solution of DS (0.5 to 3 mg/mL) under magnetic stirring (700 rpm) and mixed for 10 min. For CS/HA NPs an equal volume (0.5 mL) of an aqueous solution of CS (1 mg/mL) added to an HA aqueous solution of HA (0.5 to 4 mg/mL) were mixed at 700 rpm under magnetic stirring for 10 min and stood for 10 min.

### 2.5. Encapsulation of Peptide by Physical Entrapment Method

#### 2.5.1. CS/DS NPs

A volume (0.5 mL) of an aqueous solution of CS (1 mg/mL) containing the peptide (10 mg/mL) was added to a volume of an aqueous solution of DS (0.5 mg/mL and 3 mg/mL) and mixed at 700 rpm under magnetic stirring for 10 min. Before characterization, the formulation was stood for 10 min. The program Zetasizer 7.13 (Zetasizer ^®^ NanoZS) was used to quantify the zeta potential, polydispersity index (PDI), mean particle size (Z-average), and zeta potential. The encapsulation efficiency was calculated by determining the non-encapsulated peptide using LC/MS.

#### 2.5.2. CS/HA NPs

A volume (0.5 mL) of an aqueous solution of CS (1 mg/mL) containing the peptide (10 mg/mL) was added to a HA40 aqueous solution (1 mg/mL), and the mixture was mixed at 700 rpm while being stirred magnetically for 10 min. The encapsulation efficiency was calculated by determining the non-encapsulated peptide using LC/MS.

### 2.6. Conjugation of CS-HCl [CS] Salt and Maleimide Butyric Acid (MBA)

The main amines of CS (CS-NH_2_) and the carboxylic acid groups of maleimide butyric acid (MBA-COOH) were conjugated to create CS-MBA with the aid of systematically prepared coupling agents, N-hydroxysuccinimide (NHS) and 1-ethyl-3-(3-dimethylaminopropyl)carbodiimide (EDC). Using a proton-nuclear magnetic resonance (NMR), the degree of substitution (DS = 2.36%) was calculated. The resulting combination was dialyzed using a membrane (MWCO-7500) and the sample was sent for ^1^H-NMR analysis.

### 2.7. Conjugation of pep 1 to Modified CS-MBA

Peptides have been conjugated with modified CS-MBA, and in this instance, thiol-maleimide chemistry was used to link the peptide with the modified polymer after distinctive peaks were seen in the conjugate’s NMR spectra. CS-MBA was dissolved in an aqueous solution of 2-(N-morpholino)ethanesulfonic acid (MES) buffer 50 mM at a concentration of 12 mg/mL and to that pep 1 at a concentration of 1 mg/mL was added. It was stirred for 1 h at room temperature before being incubated from 20 to 24 h at 4 °C. The resulting mixture was next dialyzed using a membrane (MWCO-7500), and a sample was sent for ^1^H-NMR analysis using the DRX-500.

### 2.8. Conjugation of pep 2 to Modified CS-MBA

Peptides have been conjugated with modified CS-MBA by using thiol-maleimide chemistry. In a changing environment, an aqueous solution of MES buffer 50 mM was made and then conjugated CS-MBA was dissolved at a concentration of 12 mg/mL, and pep 2 was added. This mixture was stirred for 1 h at room temperature before being incubated from 20 to 24 h at 4 °C. The resulting mixture was next dialyzed using a membrane (MWCO-7500), and a sample was sent for ^1^H-NMR analysis using the DRX-500.

### 2.9. HA40 Conjugated with N-2-Aminoethyl Maleimide (AEM)

MBA was used to produce the conjugation of HA40-AEM. N-Hydroxy succinimide (NHS) and 1-(3-dimethylaminopropyl)-3-ethyl carbodiimide (EDC) were used to activate the carboxylic group of the polymer and to achieve a high degree of substitution. Conditions were tuned for the desired number of AEM conjugated with the HA40 and several degrees of substitution were tried. The following conditions were satisfied, and the resulting mixture was dialyzed using a membrane (MWCO-7500) for the first cycle with NaCl and other three cycles with water. The resultant mixture was diluted in water, and ^1^H NMR analysis was carried out using a Bruker NEO 750 spectrometer. Mestre Nova software was used in the spectral processing (MESTRELAB).

### 2.10. HA40 with AEM Conjugated with Peptides

Modified HA40-AEM has been conjugated with peptides. To do so, thiol-maleimide chemistry was employed. An aqueous solution of 4-(2-hydroxyethyl)-1-piperazineethanesulfonic acid (HEPES) buffer, 50 mM, was prepared to dissolve the HA40-AEM conjugate at a concentration of 16.4 mg/mL. To it, pep 1 (2.4 mg/mL) was added in reducing conditions adding 0.1 M tris(2-carboxyethyl)phosphine hydrochloride (TCEP). The result was a thiol-free compound, which was supplemented to avoid the formation of disulfide bonds between the peptide molecules. The resultant mixture was kept stirring for 1 h at room temperature, and then incubated for 20–24 h at 4 °C. The mixture was then dialyzed with a 7500 (MWCO) membrane (MWCO-7500) and the sample was analyzed by NMR using a Bruker DRX-500 cactus spectrometer. The same procedure was followed for pep 2 while 2.4 mg/mL concentration was used.

### 2.11. Preparation of the Nanocomplexes with CS

AEM carboxylic group was activated with EDC/NHS, and then the functionalized amine group of HA polymer was chemically conjugated. This modified polymer was then conjugated with peptide using the thiol of the peptide and the maleimide of the polymer chemistry, which produced high conjugation efficiency. Finally, by mixing CS with the modified HA-AEM-peptide, the free HA-peptide conjugate and HA-peptide/CS nanocomplexes were created.

### 2.12. NP Characterization by DLS and Nanoparticle Tracking Analysis (NTA)

Dynamic light scattering was used to describe the PDI and DLS of the NPs. After a 10-fold dilution of the NPs in ultrapure water, the zeta potential values were calculated by using laser Doppler anemometry (LDA). The Zetasizer NanoZS (Malvern Instruments, Malvern, UK) was used to test both characteristics. The observations were made at a detection angle of 173° and at a temperature of 25 °C.

### 2.13. Morphological Analysis

The NP suspension underwent morphological examination using field emission scanning electron microscopy (SEM) (Zeiss Gemini Ultra Plus, Jena, Germany). A 1:100 dilution of NPs in water was followed by a 1:10 dilution in phosphotungstic acid (2% in water). A copper grid containing carbon films was covered with the sample, which was then allowed to dry. The grids were then examined under a microscope after being cleaned with water.

### 2.14. Cells and Virus Culture

Vero cells (ATCC CCL-81, Manassas, VA, USA) were cultured in Dulbecco’s modified eagle medium (DMEM) with 4.5 g/L glucose (Microtech, Naples, Italy), 100 IU/mL penicillin, 100 g/mL streptomycin (Himedia, Naples, Italy), and 10% fetal bovine serum (Himedia). HCoV-OC43 was acquired from ATCC (VR-1558) and SARS-CoV- 2 (strain VR PV10734) was kindly donated by the Lazzaro Spallanzani Hospital of Rome, Italy. Viruses were propagated in Vero cells and maintained in small aliquots at −80 °C until use. All experimental work involving SARS-CoV-2 was performed in a biosafety level 3 (BSL3) laboratory.

### 2.15. Cytotoxicity

Compound cytotoxicity was evaluated using the 3-(4,5-dimethylthiazol-2-yl)-2,5-diphenyl-2H-tetrazolium bromide (MTT) assay (Sigma-Aldrich, St. Louis, MO, USA). In brief, Vero cells were plated in 96-well plates at a density of 2 × 10^4^ cells/well the day before the assay. Then, NPs encapsulated with peptides (VFIC and TLHC) were tested at four different concentrations (25, 50, 100, and 200 μM). After 48 h, MTT (5 mg/mL) was added to the cells and left for 3 h. Dimethyl sulfoxide (DMSO, Sigma-Aldrich) was used to dissolve the produced formazan salts. Cell viability was proportional to the activity of a mitochondrial dehydrogenase, present only in metabolically active cells, and corresponded to:

[1−(absorbance 540 nm of treated cells − absorbance 540 nm of blank/absorbance 540 nm of control cells − absorbance 540 nm of blank)] × 100

where absorbance of blank and control cells refer to the absorbance of solvent and not treated cells, respectively. The experiments were repeated three times, and the results were analyzed using GraphPad Prism software (GraphPad Software, San Diego, CA, USA).

### 2.16. Antiviral Activity

To understand whether NPs were able to inhibit coronavirus infectivity and specifically their mode of action, two different assays were performed. The difference between the two schemes of treatment is the timing of the addition of tripeptides [29].

(a) Co-treatment test. This is a screening assay to point out the antiviral compounds’ activity as antiviral agents. Cells were seeded at 2.5 × 10^5^ cells per well of a 12-well plate and cultured for 24 h at 37 °C before infection. Three duplicates of each experiment were carried out. Then, each NP modified polymer with peptide and peptide alone, was added to the cell monolayer (25–200 μM) at the same time as viral infection at a multiplicity of infection (MOI) of 0.1 plaque forming unit (pfu)/cell for 2 h at 37 °C.
(b) Virus pre-treatment. This test is useful for evaluating whether each NP modified polymer with peptide and peptide alone, can act directly on the viral particles. Each peptide was added to the virus (1 × 10^4^ pfu/mL) and incubated for 1 h at 37 °C. After incubation, the mixture (virus/peptide) was diluted on cells and incubated for two supplementary hours, so that the antiviral compounds reached a nonactive concentration and the virus was at a MOI of 0.01 pfu/cell.

The plaque test was used to determine the infectivity inhibition rate by comparing the number of plaques found in the wells treated with compounds with those found in the positive control (cells infected with virus, without treatment). At the end of each assay, cells were washed with citrate buffer (pH 3) and overlaid with carboxymethylcellulose (CMC, Sigma-Aldrich) diluted in the culture medium for 48/72 h. Then, cell monolayer was fixed with formaldehyde (Sigma-Aldrich) 4%, stained with crystal-violet (Sigma-Aldrich) solution 0.5%, and the number of plaques counted.

## 3. Results

### 3.1. Peptide Synthesis and Characterization

**Figure 1:**
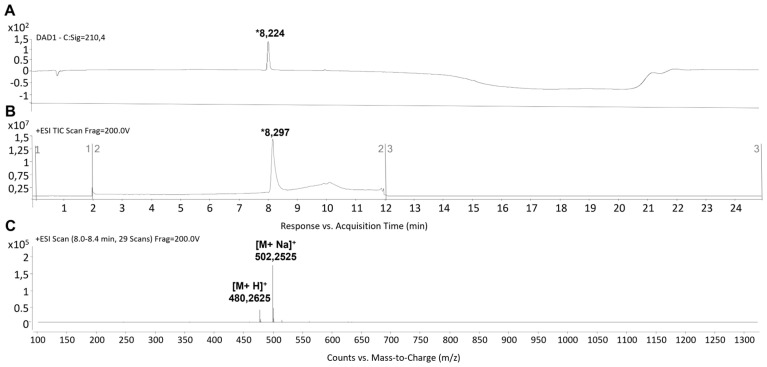
LC-MS analysis of pep 1. (**A**) Representative HPLC-DAD registered at 210 nm. (**B**,**C**) TIC and MS spectra. The tR value of the desired peptide was ~8.2 min. MS analysis showed the expected mass at *m*/*z*: 480.262 [M + H]^+^ and 502.252 [M + Na]^+^. The asterisk “*” indicates the chromatographic peak containing the target peptide.

**Figure 2:**
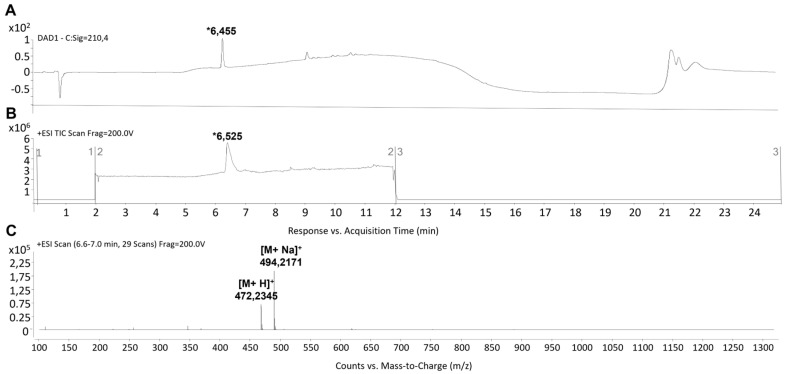
LC-MS analysis of pep 2. (**A**). Representative HPLC-DAD registered at 210 nm. (**B**,**C**) TIC and MS spectra. The tR value of the desired peptide was ~6.4 min. MS analysis showed the expected mass at *m*/*z*: 472.235 [M + H]^+^ and 494.271 [M + Na]^+^. The asterisk (*) indicates the chromatographic peak containing the target peptide. The MS values reported in the figure represent values of the main peak of the isotopic clusters.

The synthesis of peptides was carried out using the Fmoc strategy on the solid phase. The C-terminus of peptides was amidated, while the N-terminus was left free. The identity of the target peptides was confirmed through MS analysis. Detailed information on the synthesis can be found in the Materials and Methods Section. Main peaks eluting at retention times (tRs) of ~8.2 min and ~6.4 min for pep 1 and pep 2 respectively (Figure 1A,B and Figure 2A,B), were noted. Their MWs at *m*/*z* 472.235 [M + H]^+^ and 494.271 [M + Na]^+^ for pep 2, and 480.262 [M + H]^+^ and 502.252 [M + Na]^+^ for pep 1, were recorded. Accordingly, their calculated MWs were 471.21 and 479.24 g/mol for pep 2 and pep 1, respectively (Figure 1C and Figure 2C). The estimated purity of both peptides was observed to be >95%.

### 3.2. Preparation of Blank NPs and Development of Peptide-Loaded Polysaccharide NPs

As previously described by our group, the physical entrapment method for CS/DS and CS/HA NPs was used. Initially, blank NPs without peptides were prepared by simply adjusting the ionic interaction of the cationic polysaccharide (CS) with negatively charged polysaccharides (DS and HA) under mild conditions [33] (Table S1a,b). The size and zeta potential of the blank NPs were found to be within the range of acceptable values, indicating successful preparation of the blank NPs (Table 1). The mass ratio used for CS:DS was 1:0.5 and 1:3 whereas for CS:HA mass ratio of 1:3 was used to entrap the peptides. For efficient peptide encapsulation, the peptides were dissolved in the cationic phase (CS solution) according to their isoelectric point (pI) by simply adjusting the ionic interaction of the peptide and the polysaccharide (CS, DS and HA). The NPs diameter and PDI were evaluated by diffraction laser spectroscopy. Its surface was noted by electrophoretic mobility using a Zeta sizer Nano ZS90, software Zetasizer v7.13. Free peptide after ultracentrifugation was calculated by LC-MS and the encapsulation (%) was calculated as follows [34].

Encapsulation [%] = Total amount of peptide required during ultracentrifugation × 100

As shown in Table 1, the physical entrapment technique resulted in poor encapsulation of peptides (less than 10%). Hence, using the maleimide–thiol conjugation chemistry, a different strategy called conjugation strategy was developed and used further.

### 3.3. Conjugation of CS-HCl and MBA with Peptides

The conjugation of CS-MBA was accomplished by linking the primary amines of CS (CS-NH_2_) to the carboxylic acid groups of MBA-COOH through the use of coupling agents, NHS and EDC [35,36]. 1H NMR analysis led to the identification of a single peak at 6.8 ppm corresponding to the two symmetrical protons of the maleimide ring indicating desired substitution (Figure S1). The modified CS-MBA was then conjugated with peptides and in this case, thiol–maleimide chemistry was applied to link peptides with the modified polymer [37]. The NMR spectrum of conjugate exhibited characteristic peaks with specific parts per million (PPM) compared to the pure spectra of pure compounds (see Supplementary Material, Figures S2 and S3). The degree of substitution of pep 1 and pep 2 to modified CS-MBA was expected to be 90%, however 39% and 29.5%, respectively, could be recovered. Using these results, peptides were conjugated with modified polymer HA 40 kDa with the maleimide group.

### 3.4. HA40 Conjugated with AEM

**Figure 3:**
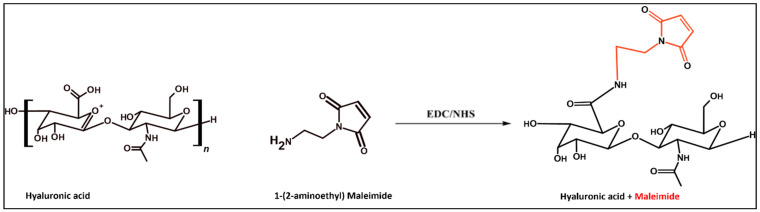
Reaction schemes of the EDC/NHS activated synthesis of HA + AEM [HA40- MAL].

**Figure 4:**
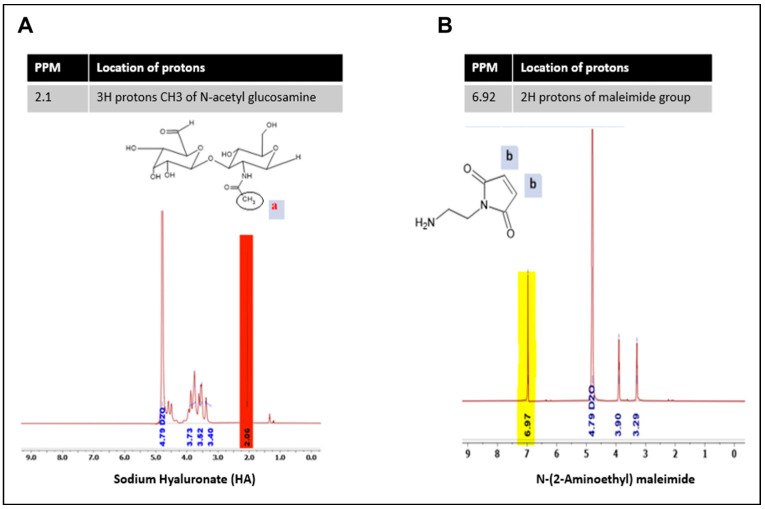
Pure spectra of (**A**) HA and (**B**) AEM. Red-colored peak indicated maleimide group, while yellow-colored peak refers to N-acetyl glucosamine of sodium hyaluronate.

**Figure 5:**
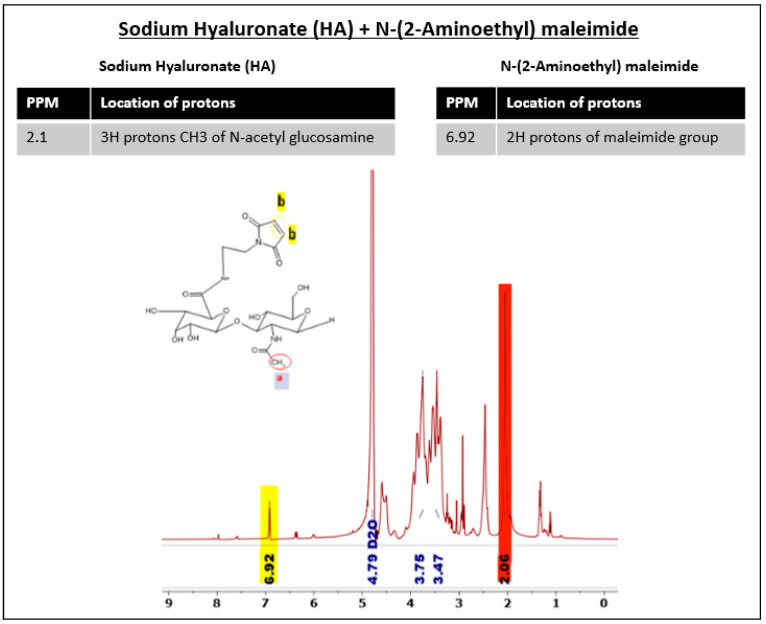
Synthesis of HA40-AEM via one-post reaction. The degree of substitution at HA 40 kDa with AEM was expected to be 20%; however, 7.428% could be recovered. Substitution the maleimide group can be observed at 6.92 ppm. Red-colored peak indicated maleimide group, while yellow-colored peak refers to N-acetyl glucosamine of sodium hyaluronate.

HA40-AEM was successfully synthesized via a one-post reaction. As schematically represented in Figure 3, the carboxylic group of the HA polymer was activated with the EDC/NHS reaction, and the functionalized amine group of AEM was chemically conjugated with it [34]. Following overnight reactions, the crude mixture solutions were purified by dialysis to remove excess of unreacted reagents and other by-products. The reaction mixture was purified by dialysis, frozen, and lyophilized after an overnight reaction. The conjugation was confirmed through ^1^H-NMR analysis, which showed a single peak at 6.8 ppm for the double bond of the maleimide and specific signal peaks for N-acetyl glucosamine at 2.1 ppm. The results are shown in Figure 4 and Figure 5.

### 3.5. Modified HA4 with AEM Was Conjugated with Peptide

**Figure 6:**
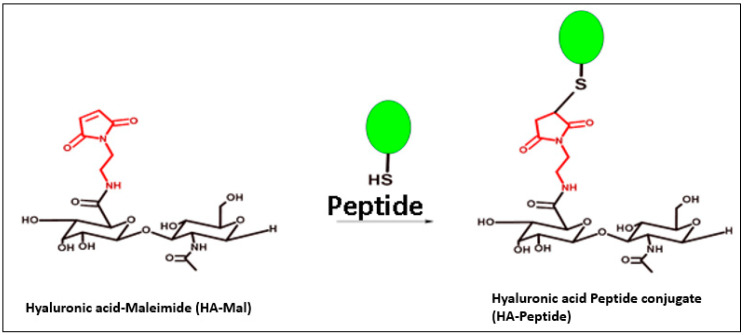
Overall systematic representation of modified polymer HA40-AEM conjugated with peptide using thiol–maleimide chemistry.

**Figure 7:**
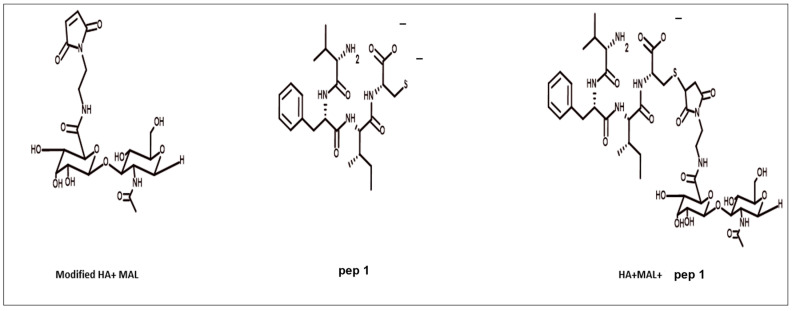
Systematic representation of modified polymer conjugated with pep 1.

**Figure 8:**
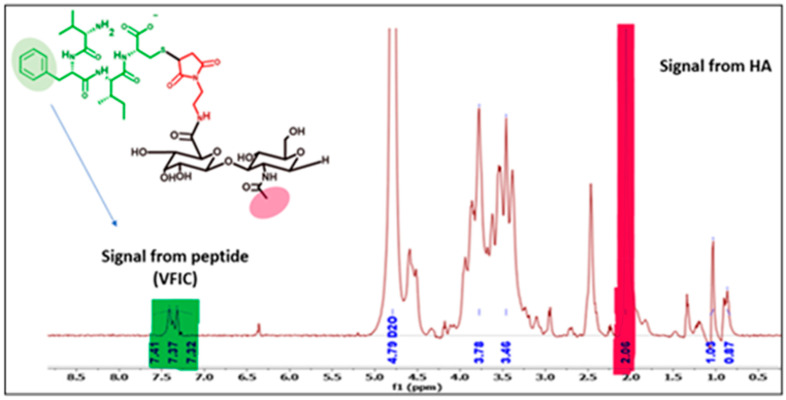
Modified HA40 with maleimide group further conjugated with pep 1. Substitution from 5H proton spectra of benzene ring was observed at 7.41, 7.37, and 7.32 ppm. A peak of HA40 at 2.1 ppm representing protons of CH_3_ of N-acetyl glucosamine was also observed.

**Figure 9:**
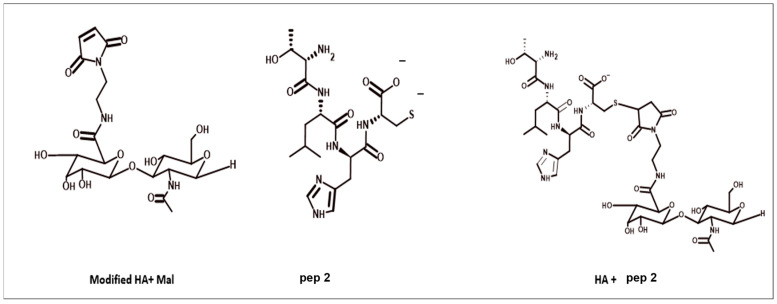
Systematic representation of the modified polymer conjugated with pep 2.

**Figure 10:**
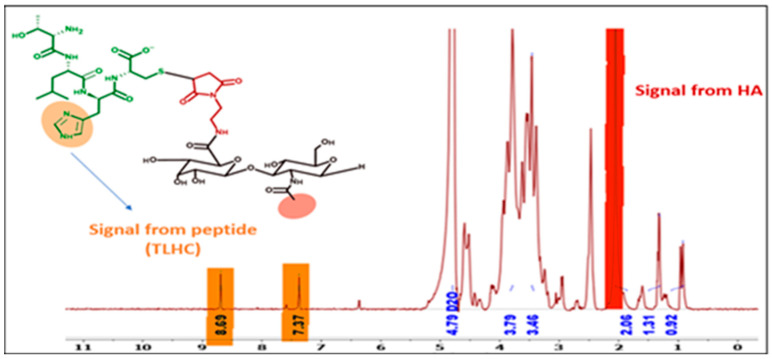
Modified HA40 with maleimide group conjugated with pep 2. Substitution occurred as seen from 1H proton spectra of histidine at 8.69 ppm and 1H proton of histidine at 7.37 ppm. Peak of HA40 at 2.1 ppm which represents protons of CH_3_ of N-acetyl glucosamine was also observed.

Modified HA40-AEM was conjugated with peptide using a thiol–maleimide chemistry as schematically represented in Figure 6 and Figure 7 [12,37,38]. The ^1^H-NMR spectrum of the conjugate exhibited characteristic peaks as evident in Figure 8, marked with arrows. An aqueous solution of 50 mM HEPES buffer was prepared to dissolve the HA40-AEM intermediate at a concentration of 16.4 mg/mL. To it, pep 1 at 2.4 mg/mL was added. Lastly, a 0.1 molar ratio of TCEP, a thiol-free compound that is highly effective in reducing peptide disulfide bonds, was added [39,40]. The resultant mixture was constantly stirred at room temperature for 1 h and then incubated for 20–24 h at 4 °C. It was then dialyzed with a membrane (MWCO-7500) and a sample was sent for ^1^H-NMR analysis using a DRX-500 spectrometer. The same procedure was repeated for the other pep 2 (Figure 9 and Figure 10).

### 3.6. Preparation and Characterization of CS/HA-Peptide NPs

CS was combined with modified HA conjugated with peptides (HA-pep 1 and HA-pep 2) to create nanocomplexes, which were characterized using DLS and NTA. The results are reported in Table 2. Low concentration of HA-peptide at around 1 mg/mL–(500 µL) and CS at 0.67 mg/mL (500 µL) resulted in small size (less than 200 nm) NPs (hereafter indicated as Small NPs). However, other NPs were produced and concentrated using an Amicon filter-30 kDa. DLS and NTA were performed pre- and post-concentration to check the disruption of NPs while no change in size was reported. The recovery of these NPs was 80–82 % as observed by lyophilizing the concentrated sample. High concentration produced larger NPs (300–400 nm) with no sample loss (hereafter indicated as Large NPs).

### 3.7. Morphological and Toxicity Analysis of CS/HA Nanoparticles Containing Peptides Linked to HA

**Figure 11:**
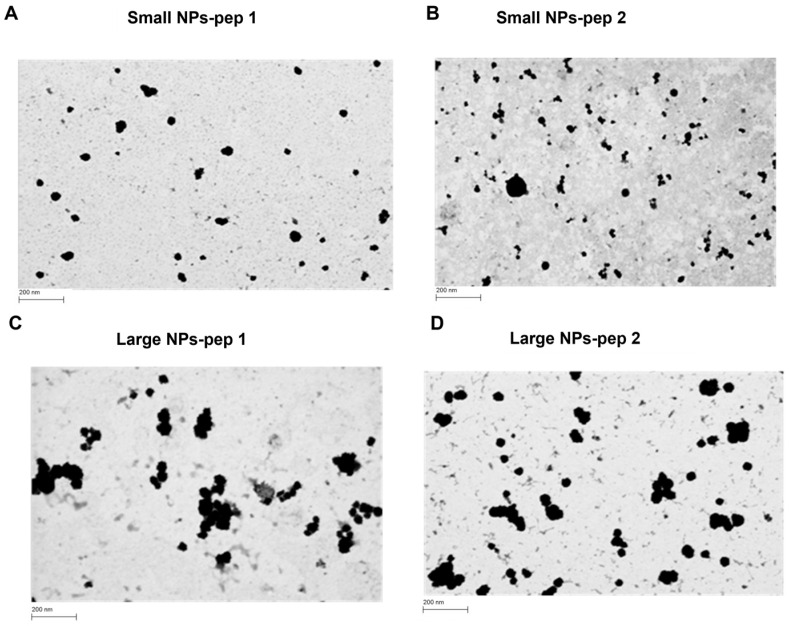
Morphological characterization of the developed nano systems. Field emission scanning electron microscopy (FESEM) images of (**A**) Small NPs-pep 1, (**B**) Small NPs-pep 2, (**C**) Large NPs-pep 1, and (**D**) Large NPs-pep 2. Scale bar: 200 nm.

**Figure 12:**
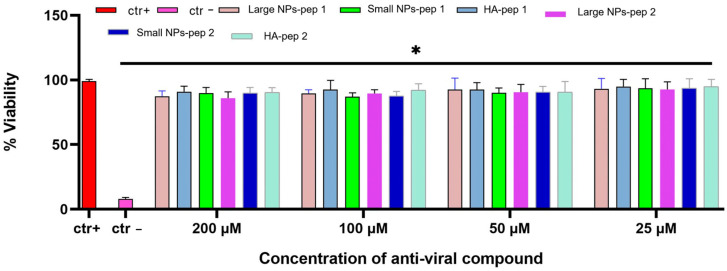
Cytotoxicity evaluation of NPs after 48 h of treatment of Vero cells. Ctr+ is medium, ctr− is DMSO. Large NPs-pep 1are NPs with high concentration of peptide, Small NPs-pep 1 are NPs with less concentration of peptide, Large NPs-pep 2 are NPs with high concentration of peptide, Small NPs-pep 2 are NPs with less concentration of peptide, HA-pep1 and HA-pep 2 are the modified polymers with peptides. * *p*  ≤  0.0001.

The shape and size of the NPs were observed through FESEM images by using STEM and InLens detectors (Figure 11). The results showed that the NPs were spherical in shape with diameters consistent with the DLS analysis values. On the other hand, as shown in Figure 12, the different prototypes showed near 100% cell viability at all the tested concentrations. The results indicated that the HA/CS nanoparticles are non-cytotoxic up to the concentration of 200 µM of each prototype. Our data are in agreement with what has been reported in the literature [41]: the HA-CS NPs caused a high increase in cell viability respect to the CS NPs in a wide range of concentrations up to 1 mg/mL. This could be explained by the agglomeration of HA-CS NPs, charactered by negative charges rendering them more stable and therefore less cytotoxic.

### 3.8. Antiviral Activity

**Figure 13:**
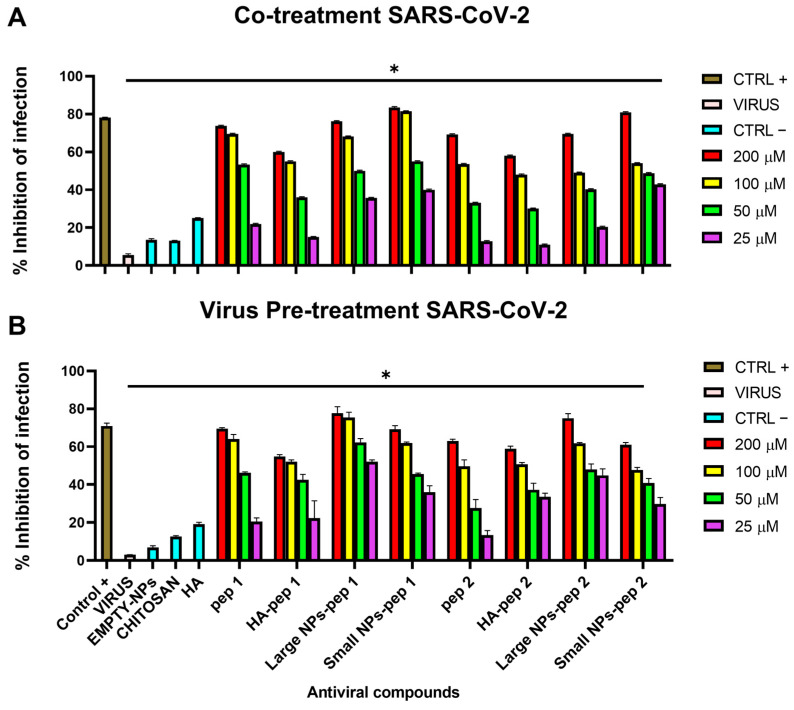
Antiviral activity of nanosystems against SARS-CoV-2. (**A**) Co-treatment assay; (**B**) virus pre-treatment assay. pep 1 and pep 2 represent only peptide and HA-pep 1 and HA-pep 2 indicate a modified polymer conjugated with peptide. Large-NPs-pep 1 and Large NPs-pep 2 are large-sized CS NPs added over a modified polymer with a high concentration of peptide. Small NPs-pep 1 and Small NPs-pep 2 are CS NPs added over a modified polymer with a low concentration of peptide. Antiparasitic drug Ivermectin was used as a positive control, while empty NP, HA and chitosan were used as negative controls. * *p*  ≤  0.0001.

**Figure 14:**
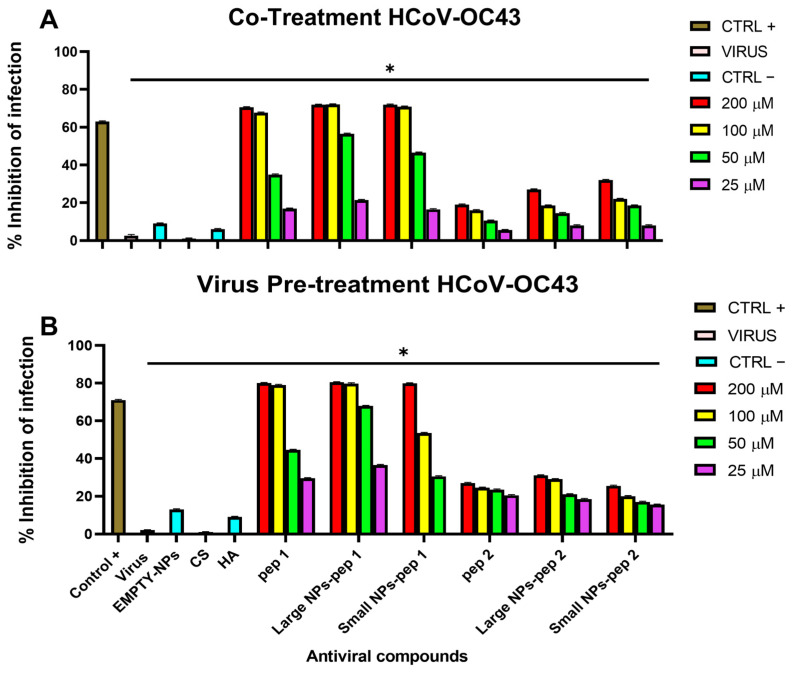
Antiviral activity of nanosystems against HCoV-OC43. (**A**) Co-treatment assay; (**B**) virus pre-treatment assay. pep 1 and pep 2 represent only peptide and HA-pep 1 and HA-pep 2 indicate a modified polymer conjugated with peptide. Large NPs-pep 1 and Large NPs-pep 2 are large-sized CS NPs added over a modified polymer with a high concentration of peptide. Small NPs-pep 1 and Small NPs-pep 2 are CS NPs added over a modified polymer with a low concentration of peptide. Antiparasitic drug Ivermectin was used as a positive control, while empty NP, HA and chitosan were used as negative controls. * *p*  ≤  0.0001.

Antiviral activity of nanocomplexes was determined via plaque assays (Figure 13 and Figure 14). Different percentages of modified polymer conjugated with peptides were also tested against virus infection (Figure S4). Two schemes of treatment were performed where NPs were added at two distinct times on cell monolayers [29]: (i) in the co-treatment test, compound and virus were simultaneously incubated on cells; (ii) in the virus pre-treatment test, compound was first added on the virus, and then the resulting mixture was diluted on cells In both the assays, the antiviral activity was dose-dependent, with increasing concentrations leading to decreased viral plaque formation and reduced infectivity [42,43]. We observed that all the NPs exhibited higher antiviral activity compared to peptide alone or modified polymer with peptide as indicated in Table 3 with the relative 50% inhibitory concentration (IC50), and it was in accordance with the morphological characteristics of NPs. However, the pep 2 derived-complexes showed little inhibition respect to the pep 1 counterparts. As expected, small-sized NPs of CS/HA containing the peptide linked to HA depicted less antiviral activity compared to big-sized NPs because of the lower yield of NPs during the concentration process (recovery 80–90%). The results showed that the large-sized NPs containing the pep 1 had better antiviral activity compared to Ivermectin used as positive control, against both SARS-CoV-2 and HCoV-OC43 in both co-treatment and virus pre-treatment tests. In addition, the antiviral activity of the pep 1 chemically linked to the HA-containing NPs, was superior than the activity of the pep 2 chemically linked to the HA-containing NPs. This results confirmed what we previously observed and could be explained by the different sites recognized by peptide on the spike protein [29].

## 4. Discussion

Infection with SARS-CoV-2 poses a major threat to the healthcare and financial sector around the world [44,45]. Despite of this, there are no effective SARS-CoV-2 therapeutic drugs. Additionally, the vaccines effectiveness for emerging COVID-19 variants ranges from 52 to 92%, which further suggests the need for effective SARS-CoV-2 therapeutic drugs [46]. In this study we found thatthe activity of AVPs can be increased through their association to nano-particles made of CS-HA. These results suggest that these NPs may have the potential to be used as a therapeutic agent against COVID-19 following intranasal administration.

Post SARS-CoV-2 infection, patients are more likely to experience pulmonary mucus hypersecretion and mucus plugging, as the mucin gene is overexpressed due to pro-inflammatory cascades [47]. This activates aryl hydrocarbon receptor signaling, resulting in increased mucus production, impaired mucociliary clearance, airway obstruction, and respiratory distress [48]. Hence, targeting mucus is a potentially viable approach for drug delivery to the site with high viral load as reflected by pathophysiological conditions. Moreover, mucus serves as a barrier between infectious pathogens and the immune system [49]. Interestingly, CS NPs have been reported to exhibit an interaction with the nasal mucosa and, therefore, may be a promising carrier for delivering AVPs directly to the virus particles entrapped in the nasopharyngeal mucosa. In addition, our group found that by conjugating biological molecules, such as mannose-derived polymers, influenza virus hemagglutinin and neuraminidase, at the surface of NPs, improved their targeting to cells presenting antigen (APCs) and cell penetration [50,51,52,53].

Peptides due to their high specificity toward the target can be delivered at relatively low concentrations for therapeutic benefits [54]. Moreover, peptide-based inhibitors show antiviral activity associated to improved safety, efficacy and higher specificity against viral pathogens [55]. Additionally, peptides are very susceptible to the surrounding conditions and become unstable if conditions are not conducive [10,54,55]. Unfavorable conditions within the human body, such as high temperature, pH, and salt concentration, cause conformational changes in peptide molecules, resulting in their inactive form [54]. Currently, different nano systems have been developed and are classified as: (i) lipid-based nanocarriers, such as liposomes; (ii) polymers, such as nanoparticles, dendrimers and micelles; (iii) inorganic nanocarriers and (iv) hybrid nanocarriers [56]. Herein, we produced nanoparticles made of CS and HA which have been shown to be promising carriers for the delivery of biological drugs and antigens [50,52,53].

CS-based NPs have the potential to enhance the antiviral effects of the drug by favoring their interaction with the nasal mucosa [57]. While using CS/HA, it was observed that β-CS depicts higher antiviral activity compared to α-CS possibly due to the parallel orientation initiating a loosely compact intermolecular hydrogen bond network. This leads to an enhanced immune response via participation of cytokine including interleukin-2, interferon-α, interferon-β, and tumor necrosis factor with an improvement in the overall efficacy of the treatment. Additionally, CS has a positive charge that may enhance the electrostatic interaction with negatively charged viral particles, thereby improving the antiviral activity. The antibacterial and antifungal activity of nano systems conjugated with peptides has been widely reported. Metal NPs conjugated with antimicrobial peptides (AMPs) showed strong effects in killing bacteria and also interfering with bacterial biofilm due to the electrostatic forces established between the cationic charge of the nano system and the negative charge of membranes [58,59,60]. In addition, metal NPs can permeate across the bacterial membrane via porins or passive diffusion and cause an efficient release of the peptide. To date, little is known of the antiviral activity of AVPs complexed with nano systems.

Interestingly, in our current study, SARS-CoV-2 and HCoV-OC43 were successfully neutralized using AVPs encapsulated in HA/CS nanocomplex. Furthermore, cytotoxicity analysis indicated that the chemical synthesized HA/CS nanocomplexes were safe carriers at concentrations up to 200 µM of nanosystem. Our data first confirmed what we already observed in our previous study [29]: pep 1-derived nano systems were endowed of higher antiviral activity against both SARS-CoV-2 and HCoV-OC43, compared to pep 2-derived polymers. This result could be explained by the different sites that the two peptides were able to recognize and bind the spike protein in two different located sites. Another important data highlighted in the present study was that large nano systems showed a stronger antiviral activity compared to small ones. This difference could be justified by a higher yield of large-sized NPs obtained during the concentration process (recovery 80–90%).

## 5. Conclusions

The results of the study showed that CS and HA conjugated with peptide NPs have strong antiviral properties and can be used as a template for the development of new antivirals against SARS-CoV-2 and HcoV-OC43. The study suggests that these NP-peptide conjugates could be potential therapeutics for treating SARS-CoV-2 and HcoV-OC43 infections.

## Figures and Tables

**Table 1:** Size, zeta potential and encapsulation efficiency calculated using LC-MS.

Nanocomplex	Z-Average Size [nm]	PDI	Zeta Potential [mv]	Encapsulation Efficiency Peptides	
				pep 1	pep 2
CS/DS NP; [CS/DS] 1:0.5 [*w*/*w*]	94 ± 5	0.152	35	Less than 10%	Less than 10%
CS/DS NP; [CS/DS] 1:3 [*w*/*w*]	168± 5	0.21	-31	Less than 10%	Less than 10%
CS/ HA NP; [CS/HA40] 1:3 [*w*/*w*]	175 ± 5	0.134	25	Less than 10%	Less than 10%

**Table 2:** Physicochemical characterization of different CS/HA nanoparticle prototypes containing the peptides linked to HA (n = 3). CS: chitosan; HA: hyaluronic acid; AEM: HA40 Conjugated with N-2-Aminoethyl Maleimide.

Description of NPs	Dynamic Light Scattering (DLS) Malvern Zetasizer [n = 3]		
	Average Size (nm)	Zeta Potential (mV)	Average Size (nm)
CS/HA-AEM-pep 1 (Small NPs-pep 1)	186 ± 2.43	22 ± 1.23	190 ± 4.39
CS/HA-AEM-pep 2 (Small NPs-pep 2)	184 ± 3.12	19 ± 1.46	180 ± 3.6
CS/HA-AEM-pep 1 (Large NPs-pep 1)	380 ± 5.1	31 ± 2.04	371 ± 7.3
CS over HA-AEM-pep 2 (Large NPs-pep 2)	420 ± 6.2	27 ± 1.96	413 ± 8.4

**Note**: Small sized NPs PDI was <0.1 and large sized NPs PDI was <0.2.

**Table 3:** IC50 of the tested compounds against SARS-CoV-2 and HCoV-OC43 in co-treatment and virus pre-treatment assays.

Compound	Co-Treatment Assay		Virus Pre-Treatment Assay	
	IC50 (μM) vs. SARS-CoV-2	IC50 (μM) vs. HCoV-OC43	IC50 (μM) vs. SARS-CoV-2	IC50 (μM) vs. HCoV-OC43
**Empty NPs**	-	-	-	-
**CS**	-	-	-	-
**HA**	-	-	-	-
**pep 1**	40	70	40	85
**HA-pep 1**	75	75	60	85
**Large NPs-pep 1**	30	30	20	30
**Small NPs-pep 1**	40	50	60	50
**pep 2**	75	-	100	-
**HA-pep 2**	75	-	85	-
**Large NPs-pep 2**	40	-	50	-
**Small NPs-pep 2**	50	-	60	-

## Data Availability

The data presented in this study are available on request from the corresponding author. Authors can confirm that all relevant data are included in the article.

## Supplementary Materials

The following supporting information can be downloaded at: https://www.mdpi.com/article/10.3390/pharmaceutics15061621/s1, Figure S1: Chitosan conjugated with maleimide and modified polymer with VFIC and TLHC; Figure S2: Conjugation of modified CS with maleimide to VFIC peptide; Figure S3: Conjugation of modified CS with maleimide THLC peptide; Figure S4: Different percentage of conjugated sodium hyaluronate with maleimide with peptide tested against SARS-CoV-2; Table S1: Screening of Blank Nanoparticles.

## References

[1] Li Q., Guan X., Wu P., Wang X., Zhou L., Tong Y., Ren R., Leung K.S.M., Lau E.H.Y., Wong J.Y. (2020). Early Transmission Dynamics in Wuhan, China, of Novel Coronavirus-Infected Pneumonia. N. Engl. J. Med..

[2] Patterson B.K., Seethamraju H., Dhody K., Corley M.J., Kazempour K., Lalezari J.P., Pang A.P., Sugai C., Francisco E.B., Pise A. (2020). Disruption of the CCL5/RANTES-CCR5 Pathway Restores Immune Homeostasis and Reduces Plasma Viral Load in Critical COVID-19. MedRxiv.

[3] Zhang H., Zhu W., Jin Q., Pan F., Zhu J., Liu Y., Chen L., Shen J., Yang Y., Chen Q. (2021). Inhalable nanocatchers for SARS-CoV-2 inhibition. Proc. Natl. Acad. Sci. USA.

[4] Hiscott J., Alexandridi M., Muscolini M., Tassone E., Palermo E., Soultsioti M., Zevini A. (2020). The global impact of the coronavirus pandemic. Cytokine Growth Factor Rev..

[5] Robinson P.C., Liew D.F.L., Tanner H.L., Grainger J.R., Dwek R.A., Reisler R.B., Steinman L., Feldmann M., Ho L.P., Hussell T. (2022). COVID-19 therapeutics: Challenges and directions for the future. Proc. Natl. Acad. Sci. USA.

[6] Weiss S.R., Leibowitz J.L. (2011). Coronavirus pathogenesis. Adv. Virus Res..

[7] Chen L., Liang J. (2020). An overview of functional nanoparticles as novel emerging antiviral therapeutic agents. Mater. Sci Eng. C Mater. Biol. Appl..

[8] Zhou X., Jiang X., Qu M., Aninwene G.E., Jucaud V., Moon J.J., Gu Z., Sun W., Khademhosseini A. (2020). Engineering Antiviral Vaccines. ACS Nano.

[9] Coen D.M., Whitley R.J. (2011). Antiviral drugs and antiviral drug resistance. Curr. Opin. Virol..

[10] Corsi F., Sorrentino L., Mazzucchelli S., Truffi M., Capetti A., Rizzardini G., Fiandra L. (2016). Antiretroviral therapy through barriers: A prominent role for nanotechnology in HIV-1 eradication from sanctuaries. J. Pharm. Pharmacol..

[11] Fontana R.J. (2009). Side effects of long-term oral antiviral therapy for hepatitis B. Hepatology.

[12] Hensel A., Bauer R., Heinrich M., Spiegler V., Kayser O., Hempel G., Kraft K. (2020). Challenges at the Time of COVID-19: Opportunities and Innovations in Antivirals from Nature. Planta Med..

[13] Delshadi R., Bahrami A., McClements D.J., Moore M.D., Williams L. (2021). Development of nanoparticle-delivery systems for antiviral agents: A review. J. Control. Release.

[14] Mahendran A.S.K., Lim Y.S., Fang C.M., Loh H.S., Le C.F. (2020). The Potential of Antiviral Peptides as COVID-19 Therapeutics. Front. Pharm..

[15] Karoyan P., Vieillard V., Gomez-Morales L., Odile E., Guihot A., Luyt C.E., Denis A., Grondin P., Lequin O. (2021). Human ACE2 peptide-mimics block SARS-CoV-2 pulmonary cells infection. Commun. Biol..

[16] Zhou Y., Simmons G. (2012). Development of novel entry inhibitors targeting emerging viruses. Expert Rev. Anti Infect. Ther..

[17] Chastain D.B., Stover K.R., Riche D.M. (2017). Evidence-based review of statin use in patients with HIV on antiretroviral therapy. J. Clin. Transl. Endocrinol..

[18] de Leuw P., Stephan C. (2018). Protease inhibitor therapy for hepatitis C virus-infection. Expert Opin. Pharm..

[19] Sulkowski M.S. (2003). Hepatotoxicity associated with antiretroviral therapy containing HIV-1 protease inhibitors. Semin Liver Dis..

[20] Tsantrizos Y.S. (2008). Peptidomimetic therapeutic agents targeting the protease enzyme of the human immunodeficiency virus and hepatitis C virus. Acc. Chem. Res..

[21] Hayden F.G., Shindo N. (2019). Influenza virus polymerase inhibitors in clinical development. Curr. Opin. Infect. Dis..

[22] Meng J., Agrahari V., Ezoulin M.J., Zhang C., Purohit S.S., Molteni A., Dim D., Oyler N.A., Youan B.C. (2016). Tenofovir Containing Thiolated Chitosan Core/Shell Nanofibers: In Vitro and in Vivo Evaluations. Mol. Pharm..

[23] McClements D.J. (2018). Encapsulation, protection, and delivery of bioactive proteins and peptides using nanoparticle and microparticle systems: A review. Adv. Colloid Interface Sci..

[24] Calvo P., Remuñan-López C., Vila-Jato J.L., Alonso M.J. (1997). Chitosan and chitosan/ethylene oxide-propylene oxide block copolymer nanoparticles as novel carriers for proteins and vaccines. Pharm Res..

[25] Fernández Urrusuno R., Calvo P., Remuñán-López C., Vila Jato J.L., Alonso M.J. (1999). Enhancement of nasal absorption of insulin using chitosan nanoparticles. Pharm. Res..

[26] Behrens I., Pena A.I., Alonso M.J., Kissel T. (2002). Comparative uptake studies of bioadhesive and non-bioadhesive nanoparticles in human intestinal cell lines and rats: The effect of mucus on particle adsorption and transport. Pharm Res..

[27] Vila A., Sánchez A., Janes K., Behrens I., Kissel T., Vila Jato J.L., Alonso M.J. (2004). Low molecular weight chitosan nanoparticles as new carriers for nasal vaccine delivery in mice. Eur J Pharm Biopharm..

[28] Köping-Höggård M., Sánchez A., Alonso M.J. (2005). Nanoparticles as carriers for nasal vaccine delivery. Expert Rev Vaccines..

[29] Zannella C., Chianese A., Greco G., Santella B., Squillaci G., Monti A., Doti N., Sanna G., Manzin A., Morana A. (2022). Design of Three Residues Peptides against SARS-CoV-2 Infection. Viruses.

[30] Mohammed M., Devnarain N., Elhassan E., Govender T. (2022). Exploring the applications of hyaluronic acid-based nanoparticles for diagnosis and treatment of bacterial infections. Wiley Interdiscip. Rev. Nanomed. Nanobiotechnol..

[31] Jaber N., Al-Remawi M., Al-Akayleh F., Al-Muhtaseb N., Al-Adham I.S.I., Collier P.J. (2022). A review of the antiviral activity of Chitosan, including patented applications and its potential use against COVID-19. J. Appl. Microbiol..

[32] Caporale A., Doti N., Monti A., Sandomenico A., Ruvo M. (2018). Automatic procedures for the synthesis of difficult peptides using oxyma as activating reagent: A comparative study on the use of bases and on different deprotection and agitation conditions. Peptides.

[33] Li H., Nykoluk M., Li L., Liu L.R., Omange R.W., Soule G., Schroeder L.T., Toledo N., Kashem M.A., Correia-Pinto J.F. (2017). Natural and cross-inducible anti-SIV antibodies in Mauritian cynomolgus macaques. PLoS ONE.

[34] Nair D.P., Podgórski M., Chatani S., Gong T., Xi W., Fenoli C.R., Bowman C.N. (2014). The Thiol-Michael Addition Click Reaction: A Powerful and Widely Used Tool in Materials Chemistry. Chem. Mater..

[35] Fernández-Urrusuno R., Romani D., Calvo P., Vila-jato J.L., Alonso M.J. (1999). Development of a freeze-dried formulation of insulin-loaded chitosan nanoparticles intended for nasal administration. STP Pharma Sci..

[36] Totaro K.A., Liao X., Bhattacharya K., Finneman J.I., Sperry J.B., Massa M.A., Thorn J., Ho S.V., Pentelute B.L. (2016). Systematic Investigation of EDC/Snhs-Mediated Bioconjugation Reactions for Carboxylated Peptide Substrates. Bioconjug. Chem..

[37] Matsumoto M., Udomsinprasert W., Laengee P., Honsawek S., Patarakul K., Chirachanchai S. (2016). A Water-Based Chitosan-Maleimide Precursor for Bioconjugation: An Example of a Rapid Pathway for an In Situ Injectable Adhesive Gel. Macromol. Rapid Commun..

[38] Smyth D.G., Nagamatsu A., Fruton J.S. (1960). Some Reactions of N-Ethylmaleimide1. J. Am. Chem. Soc..

[39] Baldwin A.D., Kiick K.L. (2011). Tunable degradation of maleimide-thiol adducts in reducing environments. Bioconjug. Chem..

[40] Madler S., Bich C., Touboul D., Zenobi R. (2009). Chemical cross-linking with NHS esters: A systematic study on amino acid reactivities. J. Mass Spectrom..

[41] Almalik A., Alradwan I., Majrashi M.A., Alsaffar B.A., Algarni A.T., Alsuabeyl M.S., Alrabiah H., Tirelli N., Alhasan A.H. (2018). Cellular responses of hyaluronic acid-coated chitosan nanoparticles. Toxicol. Res..

[42] Nielsen J.S., Buczek P., Bulaj G. (2004). Cosolvent-assisted oxidative folding of a bicyclic alpha-conotoxin ImI. J. Pept. Sci..

[43] Thong Q.X., Wong C.L., Ooi M.K., Kueh C.L., Ho K.L., Alitheen N.B., Tan W.S. (2018). Peptide inhibitors of Macrobrachium rosenbergii nodavirus. J. Gen. Virol..

[44] Zannella C., Giugliano R., Chianese A., Buonocore C., Vitale G.A., Sanna G., Sarno F., Manzin A., Nebbioso A., Termolino P. (2021). Antiviral Activity of Vitis vinifera Leaf Extract against SARS-CoV-2 and HSV-1. Viruses.

[45] Zannella C., Chianese A., Palomba L., Marcocci M.E., Bellavita R., Merlino F., Grieco P., Folliero V., De Filippis A., Mangoni M. (2022). Broad-Spectrum Antiviral Activity of the Amphibian Antimicrobial Peptide Temporin L and Its Analogs. Int. J. Mol. Sci..

[46] Campora S., Ghersi G. (2022). Recent developments and applications of smart nanoparticles in biomedicine. Nanotechnol. Rev..

[47] Kaye A.D., Okeagu C.N., Pham A.D., Silva R.A., Hurley J.J., Arron B.L., Sarfraz N., Lee H.N., Ghali G.E., Gamble J.W. (2021). Economic impact of COVID-19 pandemic on healthc.care facilities and systems: International perspectives. Best Pr. Res. Clin. Anaesthesiol..

[48] https://www.cdc.gov/media/releases/2022/s0715-COVID-VE.html.

[49] Vainshelboim B. (2021). Retracted: Facemasks in the COVID-19 era: A health hypothesis. Med. Hypotheses.

[50] Alonso-Sande M., Teijeiro-Osorio D., Remuñán-López C., Alonso M.J. (2009). Glucomannan, a promising polysaccharide for biopharmaceutical purposes. Eur. J. Pharm. Biopharm..

[51] Wattendorf U., Coullerez G., Vörös J., Textor M., Merkle H.P. (2008). Mannose-based molecular patterns on stealth microspheres for receptor-specific targeting of human antigen-presenting cells. Langmuir.

[52] Csaba N., Garcia-Fuentes M., Alonso M.J. (2009). Nanoparticles for nasal vaccination. Adv. Drug Deliv. Rev..

[53] Samaridou E., Alonso M.J. (2018). Nose-to-brain peptide delivery—The potential of nanotechnology. Bioorg Med Chem..

[54] Gulati N., Dua K., Dureja H. (2021). Role of chitosan based nanomedicines in the treatment of chronic respiratory diseases. Int. J. Biol. Macromol..

[55] Stan D., Enciu A.M., Mateescu A.L., Ion A.C., Brezeanu A.C., Stan D., Tanase C. (2021). Natural Compounds With Antimicrobial and Antiviral Effect and Nanocarriers Used for Their Transportation. Front. Pharmacol..

[56] Zhang W., Ronca S., Mele E. (2017). Electrospun Nanofibres Containing Antimicrobial Plant Extracts. Nanomaterials.

[57] Tan R.S.L., Hassandarvish P., Chee C.F., Chan L.W., Wong T.W. (2022). Chitosan and its derivatives as polymeric anti-viral therapeutics and potential anti-SARS-CoV-2 nanomedicine. Carbohydr. Polym..

[58] Mohanty S., Jena P., Mehta R., Pati R., Banerjee B., Patil S., Sonawane A. (2013). Cationic antimicrobial peptides and biogenic silver nanoparticles kill mycobacteria without eliciting DNA damage and cytotoxicity in mouse macrophages. Antimicrob Agents Chemother..

[59] Rai A., Pinto S., Velho T.R., Ferreira A.F., Moita C., Trivedi U., Evangelista M., Comune M., Rumbaugh K.P., Simões P.N. (2016). One-step synthesis of high-density peptide-conjugated gold nanoparticles with antimicrobial efficacy in a systemic infection model. Biomaterials..

[60] Casciaro B., Moros M., Rivera-Fernández S., Bellelli A., de la Fuente J.M., Mangoni M.L. (2017). Gold-nanoparticles coated with the antimicrobial peptide esculentin-1a(1-21)NH2 as a reliable strategy for antipseudomonal drugs. Acta Biomater..

