# Supplementary material for: Antiviral Peptides Delivered by Chitosan-Based Nanoparticles to Neutralize SARS-CoV-2 and HCoV-OC43
Source: Pharmaceutics. 2023 May 30;15(6):1621. doi: 10.3390/pharmaceutics15061621 (PMC10305280; doi:10.3390/pharmaceutics15061621)
Supplement: Supplementary file 1 [file pharmaceutics-15-01621-s001.zip › pharmaceutics-2283213-supplementary.pdf]

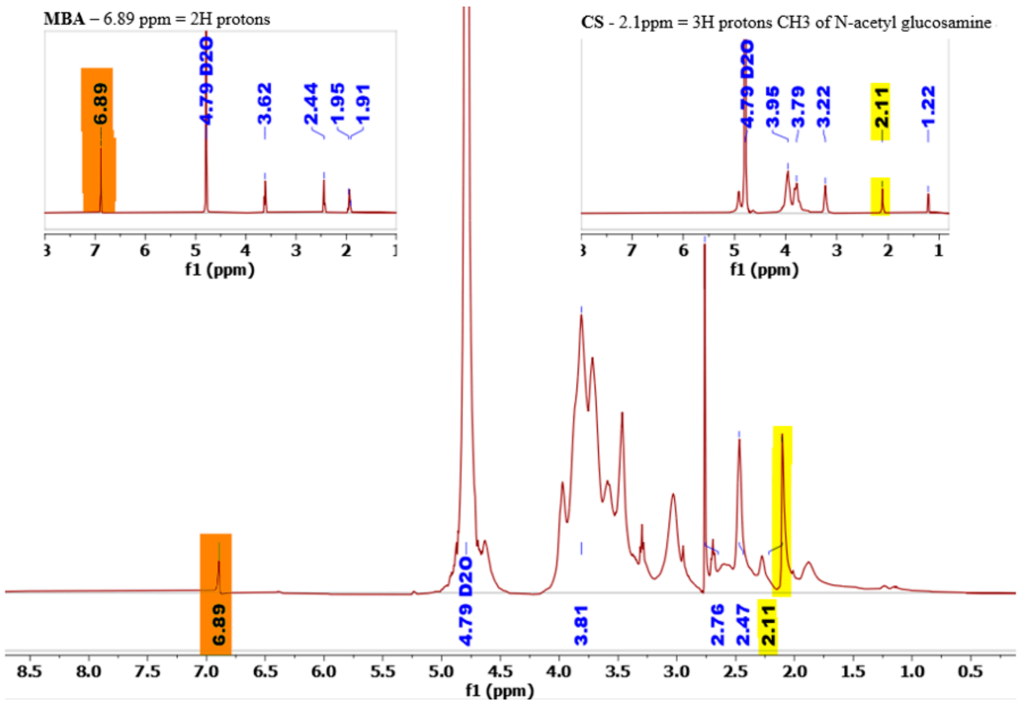

Figure S1: Chitosan conjugated with Malemide and Modified polymer with pep 1 and pep 2. Conjugation of chitosan to maleimide was achieved has substitution occurred as it is demonstrated by peak 6.89 ppm (Right hand side pure NMR spectra of Maleimide butyric acid and left-hand side pure spectra of chitosan between both spectra illustration of conjugated chitosan to maleimide) CS- 2.1 ppm= 3H protons CH_3 of N-acetyl glucosamine and MBA- 6.89 ppm= 2H protons

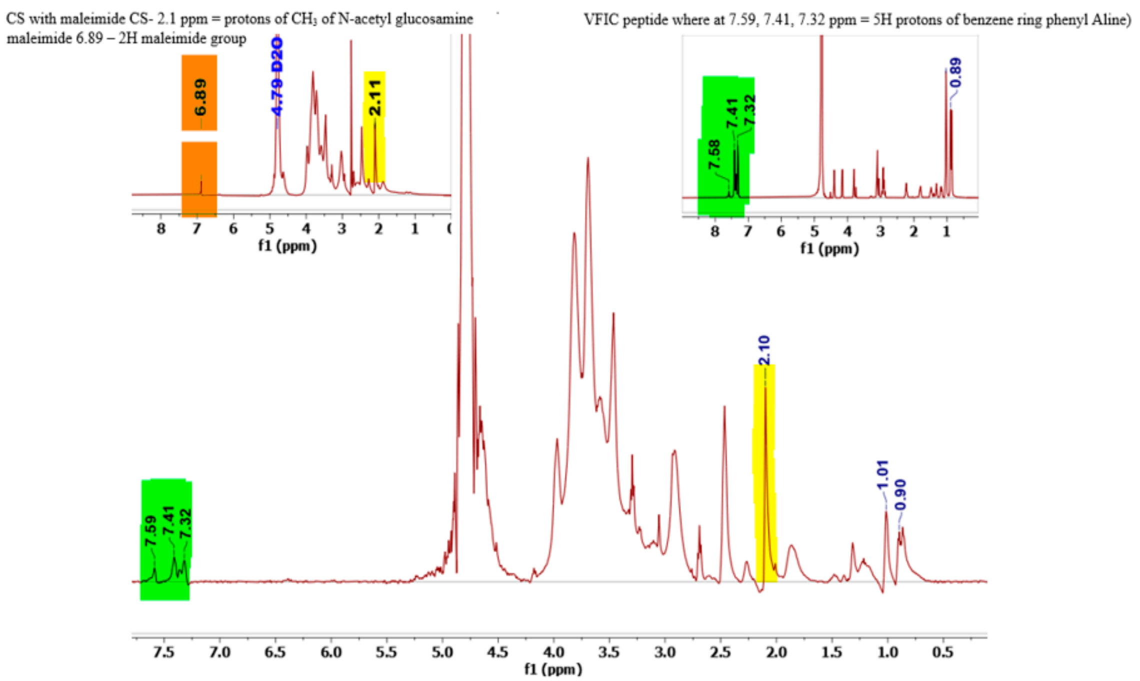

Figure S2: Conjugation of modified CS with maleimide to pep 1. Substitution occurred at 5H protons of benzene ring phenyl aline peaks, observed at 7.59, 7.41, 7.32 ppm. (Right hand side pure spectra of modifies CS with maleimide CS- 2.1 ppm = protons of CH_3 of N-acetyl glucosamine and MBA-6.89 -2H malemide group and left hand side pure spectra of pep 1- 7.59, 7.41, 7.32 ppm=5H protons of benzene ring phenyl aline.

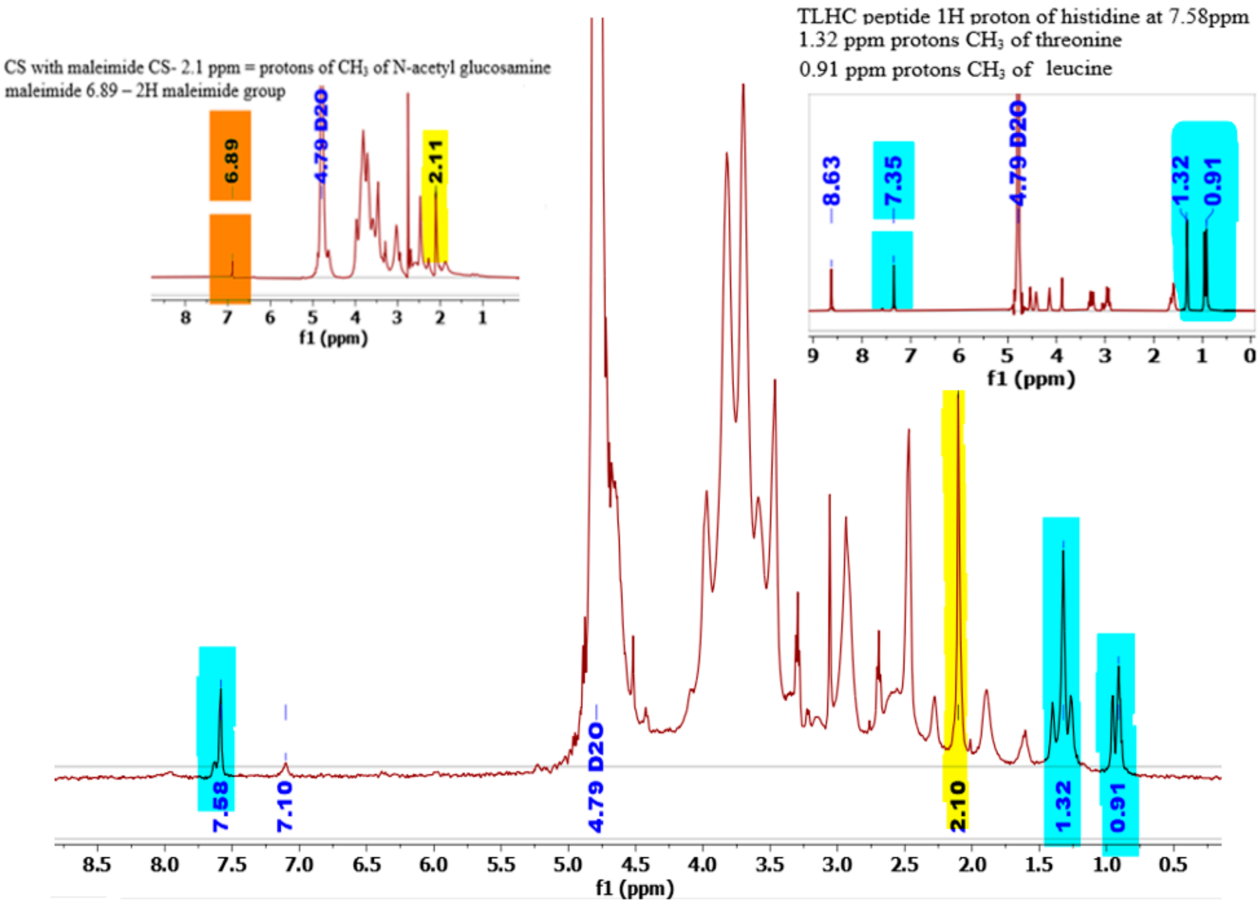

Figure S3: Conjugation of modified CS with maleimide pep 2. Substitution occurred at 1H proton of histidine at 7.58 ppm and 1.32 ppm protons CH_3 of theonine, 0.91 ppm protons CH_3 of leucine and there are also peaks of CS-2.1 ppm = protons of CH_3 of N-acetyl glucosamine (right hand side CS with malemide CS-2.1 ppm = protons of CH_3 of N-acetyl glucosamine and MBA-6.89 -2H malemide group and left hand side pep 2 at 1H proton of histidine at CH_3 of theonine, 0.91 ppm protons CH_3 of leucine).

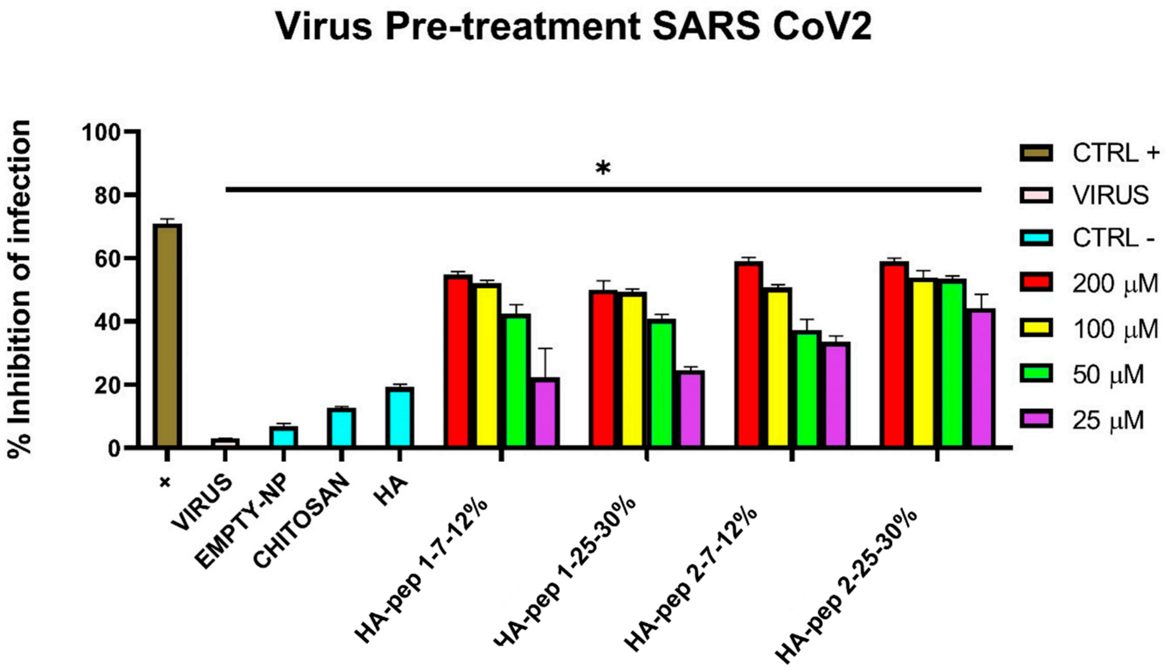

Figure S4: Different percentage of conjugated sodium hyaluronate with Malemide with peptide was tested against SARS-CoV-2. * $p \leq 0.0001$.

Table S1: Screening of Blank Nanoparticles

(a) **Chitosan over Dextran sulphate [CS/DS]**				
**N=3 triplicate of each sample was done**				
**Chitosan over Dextran sulphate [CS/DS]**		**Z-Average size [nm]**	**PDI**	**Zeta Potential [mv]**
**Mass ratio [w/w]**	**Charge ratio [+/-]**			
1:0.5		125 ± 5	0.152	35
1:1		137 ± 5	0.184	-29
1:2		72 ± 5	0.213	-33
1:3		68± 5	0.21	-31

(b) **Chitosan over sodium hyaluronic acid [CS/HA40]**				
**N=3 triplicate of each sample was done**				
**Chitosan over sodium hyaluronic acid [CS/HA40]**		**Z-Average size [nm]**	**PDI**	**Zeta Potential [mv]**
**Mass ratio [w/w]**	**Charge ratio [+/-]**			
1:1 [w/w]		175 ± 5	0.134	25
1:2 [w/w]		719 ± 5	0.431	-19
1:3 [w/w]		192± 5	0.091	-31
1:4 [w/w]		231± 5	0.068	-33

One positive zeta potential nanoparticles with mass ratio [CS/DS 1:0.5] and one negative zeta potential nanoparticles with mass ratio [CS/DS 1:3] were selected for encapsulation of antiviral peptides of polymeric nanoparticles.
